# An Isobaric Labeling Approach to Enhance Detection and Quantification of Tissue-Derived Plasma Proteins as Potential Early Disease Biomarkers

**DOI:** 10.3390/biom13020215

**Published:** 2023-01-22

**Authors:** Sumaiya Nazli, Kip D. Zimmerman, Angelica M. Riojas, Laura A. Cox, Michael Olivier

**Affiliations:** 1 Center for Precision Medicine, Wake Forest University School of Medicine, Winston-Salem, NC 27157, USA; 2 Department of Internal Medicine, Section on Molecular Medicine, Wake Forest University School of Medicine, Winston-Salem, NC 27157, USA

**Keywords:** proteomics, mass spectrometry, tandem mass tag (TMT), biomarker, plasma proteomics

## Abstract

The proteomic analysis of plasma holds great promise to advance precision medicine and identify biomarkers of disease. However, it is likely that many potential biomarkers circulating in plasma originate from other tissues and are only present in low abundances in the plasma. Accurate detection and quantification of low abundance proteins by standard mass spectrometry approaches remain challenging. In addition, it is difficult to link low abundance plasma proteins back to their specific tissues or organs of origin with confidence. To address these challenges, we developed a mass spectrometry approach based on the use of tandem mass tags (TMT) and a tissue reference sample. By applying this approach to nonhuman primate plasma samples, we were able to identify and quantify 820 proteins by using a kidney tissue homogenate as reference. On average, 643 ± 16 proteins were identified per plasma sample. About 58% of proteins identified in replicate experiments were identified both times. A ratio of 50 μg kidney protein to 10 μg plasma protein, and the use of the TMT label with the highest molecular weight (131) for the kidney reference yielded the largest number of proteins in the analysis, and identified low abundance proteins in plasma that are prominently found in the kidney. Overall, this methodology promises efficient quantification of plasma proteins potentially released from specific tissues, thereby increasing the number of putative disease biomarkers for future study.

## 1. Introduction

The promise of precision medicine for improved patient care and disease management critically depends on early disease biomarker signatures that predict health outcomes, facilitate early diagnosis, and inform treatment [1]. Ideally, individual biomarkers or biomarker signatures composed of multiple biomarkers can be assayed accurately, quickly, and cost-effective using minimally invasive approaches. It is for this reason that biomarker studies have often focused on the analysis of blood or other easily collected body fluids. Examples of commonly used clinical plasma biomarkers include the analysis of liver-specific enzymes such as alkaline phosphatase (ALP), alanine transaminase (ALT), aspartate transaminase (AST), or gamma-glutamyl transferase (GGT) for liver function and dysfunction [2,3,4,5,6,7], or the detection of troponin I as a biomarker for myocardial damage [8,9,10]. Nonetheless, protein concentrations in plasma span many orders of magnitude, and many proteins that are released from tissues and organs into the circulation are only present in low concentrations [11,12]. In addition, the tissue or cell type of origin for these “tissue leakage proteins” is often difficult to ascertain, challenging the usefulness of these proteins as biomarkers for early disease or tissue damage caused by disease progression. 

Over the past two decades, liquid chromatography-coupled tandem mass spectrometry (MS) has become the primary methodology for untargeted proteomics analysis, including biomarker discovery. In a standard workflow, peptides go through two levels of MS analysis [13,14,15]. The initial MS scan (MS1) determines the mass to charge proportion (*m*/*z*) for each ion that is detected in the sample. Subsequently, individual peptide ions are fragmented and a fragment MS spectrum (MS2) is collected that aids in the determination of the amino acid sequence of the initial peptide [16]. Low abundance peptide ions often generate MS2 fragment spectra that make identification of the original peptide challenging. This challenge in identifying low-abundance proteins is further exacerbated in samples such as plasma where extremely highly abundant proteins impede the MS detection of lower abundance proteins. About 55% of the total protein mass in plasma is attributable to albumin alone, and the most abundant seven proteins together account for 85% of the total protein mass [17]. Therefore, depletion methodologies have been developed to reduce the fraction of these abundant proteins, such as the MARS-14 columns (Agilent) or the High Select Top 14 Abundant Protein Depletion Mini Spin Columns (Thermo) that both reduce the target protein abundance by up to 95%, without affecting the global protein expression patterns of the samples [17]. These depletion approaches facilitate the detection of lower abundance proteins by mass spectrometry.

Tandem mass tag (TMT) labeling is a widely used technique in proteomics for the identification and quantification of proteins [18,19]. TMTs are isobaric labeling compounds that create unique reporter ions during mass spectrometry. The labels are comprised of a MS/MS reporting group, a spacer arm, and an amine reactive group. The amine reactive group is attached to the N-terminus or lysine side chain of a peptide [20]. In relative quantitation analyses, diverse isobaric labels are utilized to label proteins from individual samples with different tags. Once labeled, individual samples are combined and run in a liquid chromatography-mass spectrometry (LC-MS) analysis [20]. Since the isobaric labels have essentially identical properties, all peptides from different samples with different labels co-elute during LC separation. Peptides are identified as a single precursor ion peak in MS1, and the reporter ions are released during MS2 fragmentation, allowing the relative quantification of each peptide in the different samples. These reporter ions are detected with much higher sensitivity than peptide ions, and it is this property that has been exploited in the analysis of extremely low abundance proteins in single cell proteomics. In the SCoPE-MS (Single Cell ProtEomics by Mass Spectrometry) approach, proteins isolated from individual cells are labeled with specific TMT tags, and mixed with a reference sample consisting of proteins isolated from a pool of several hundred cells, labeled with a different TMT tag [21]. The abundance of individual proteins and peptides in a single cell is mostly below the detection limit of mass spectrometers, and do not generate a sufficient number of fragment ions during MS2 to ensure confident identification of the peptide. During MS analysis in SCoPE-MS, the peptide identification and characterization is driven by the more abundant proteins in the pool of reference sample; however, TMT reporter ions can be detected for each peptide for both the pool of reference sample and the individual cells. 

In this study, we describe an adaptation of this approach for the identification of low-abundance tissue leakage proteins in plasma. With this approach, any peptide identified during MS analysis in a tissue reference sample can subsequently be quantified in individual plasma samples using the TMT reporter ions, and would be detected even if present in low amounts. This mass spectrometry plasma proteomics methodology to discover novel disease markers will expand our ability to detect proteins from specific tissues in plasma samples, and expand the list of putative tissue-derived biomarker proteins for many diseases and tissues.

## 2. Materials and Methods

### 2.1. Samples

All samples used in this study were collected from female olive baboons (*Papio hamadrayas)* that were part of a pedigreed baboon colony from the Southwest National Primate Research Center at the Texas Biomedical Research Institute, San Antonio, Texas [12]. Plasma and kidney samples were collected under a protocol approved by the Institute’s Institutional Animal Care and Use Committee (IACUC). 

### 2.2. Methods

*Sample Preparation*: Baboon kidney medulla and cortex biopsies were collected and flash frozen in liquid nitrogen and stored at −80 °C. Frozen tissue aliquots were isolated under a sterile laminar flow hood using sterile scalpels and tweezers. Aliquots of approximately 2 mg were collected on a frozen aluminum plate in dry ice and transferred to bead tube with 500 uL Tris HCl on ice for homogenization. Homogenization was completed by bead beating in a frozen metal tube holder for 30 seconds at 2000 rpm. About 10 uL of plasma was used with the Thermo Scientific High-Select Top14 Abundant Protein Depletion kit (Catalog number: A36369, Thermo Fisher Scientific, Waltham, MA, USA) for removal of the 14 highly abundant proteins. 

Both plasma and homogenized tissues were precipitated using acetone. For unlabeled proteomics (label-free proteomics, LFQ), 100 μg of the resulting tissue proteins were dissolved in 100 mM ammonium bicarbonate (ABC) (Catalog number: BB-2603, Boston BioProducts, Ashland, MA, USA) buffer and followed by three major steps: 25 mM dithiothreitol (DTT) (Catalog number: A39255, Thermo Fisher Scientific, Waltham, MA, USA) for reduction, 13.875 mM iodoacetamide (IAA) (Catalog number: A39271, Thermo Fisher Scientific, Waltham, MA, USA) for acetylation, and 2.5 μg trypsin for overnight digestion. The digested samples were further fractionated using the Thermo pH fractionation kit (Pierce High pH Reversed-Phase Peptide Fractionation). 

*Mass Spectrometry:* For TMT proteomics, 100 μg of total precipitated proteins from kidney cortex, medulla, and plasma in 100 mM TEAB were processed using 200 mM TCEP, 375 mM IAA, and 2.5 μg trypsin digestion. Cortex, medulla, and plasma digests were labeled with the designated TMT10plex channel (TMT10plex™ Isobaric Label Reagent Set, 3 × 0.8 mg, Catalog number: 90111, ThermoFisher Scientific, Waltham, MA, USA) reagents (see Supplementary Table S1 for specific information) and combined according to ratios of kidney to plasma protein concentrations outlined in Table 1. For example, a ratio of 5:1 (kidney tissue to plasma) contains 50 μg peptides from each tissue sample to 10 μg peptides from each plasma sample. A blank TMT channel was placed following the kidney tissue prior to the channels containing plasma to limit interference of reporter ion intensities abundant proteins from the kidney samples. Multiplexed samples were processed further using Thermo pH fractionation kit (Pierce High pH Reversed-Phase Peptide Fractionation, Catalog number: PI84868, ThermoFisher Scientific, Waltham, MA). 

After fractionation, eight fractions were pooled to four (fraction 1 to 5, 2 to 6, etc.,) and 1 μg of each sample was loaded on a PepMap RSLC C18 easy-spray column (3 μm, 100 A, 75 μm × 15 cm) using Easy-nLC 1200 coupled to an Orbitrap Lumos Tribrid Mass Spectrometer (ThermoFisher Scientific, Waltham, MA, USA). Peptides were separated using a 2-h gradient of Mobile phase A (0.1% formic acid in 95:5 water: acetonitrile) and Mobile Phase B (0.1% formic acid in 80:20 acetonitrile: water). The following gradient program was used for peptide elution: 2–30% B in 85 minutes, 30–95% B in 30 minutes, and 95% B in 5 minutes. Data were acquired in MS1 scan mode (*m*/*z* = 400–1600) at a resolution of 120,000 with automatic gain control (AGC) of 1 × 10^6^ and maximum injection time of 50 ms. MS/MS data acquisition was done using higher-energy C-trap dissociation (HCD) mode in Orbitrap detection at a resolution of 50,000 with an AGC target of 1.25 × 10^5^ and maximum injection time of 86 ms. All data acquisition was carried out using Thermo Scientific Xcalibur software (software version Xcalibur 4.3, ThermoFisher Scientific, Waltham, MA, USA) and data analysis was completed in MaxQuant (software version MaxQuant 2.1.1.0, https://www.maxquant.org/, accessed on 9 January 2023 ) against *P. anubis* proteome database (*P. anubis* Uniprot Reference Proteome ID UP000028761, 43,406 protein entries) using the default parameters for TMT or LFQ analysis. Modifications were set to the default variable modifications (oxidation (M) and acetyl (protein N-term)) and fixed modifications (carbamidomethyl). Individual peptide mass tolerance was selected to filter peptides according to individual peptide mass tolerances. False discovery rates (FDR) were set to 0.01 at both the PSM and the protein level. MaxQuant output files (proteingroups) were manually curated, and contaminant matches or protein identifications with no measured reporter ion intensities across all samples were removed. All results files can be found in the Supplementary Tables S2–S11.

## 3. Results

As described in the Methods section, we used nonhuman primate baboon (Papio hamadrayas) samples for development and optimization of our method. Seven plasma samples were collected as part of a recent study [22] and kidney cortex and medulla samples from a healthy animal were collected at necropsy. An initial label-free analysis using mass spectrometry identified on average 106 proteins in a plasma sample (range 79–135), and 489 and 1780 proteins from the kidney cortex and medulla, respectively (Table 2). A complete list of the identified proteins can be found in Supplementary Tables S10 (for plasma samples) and S11 (for kidney samples).

We implemented an isotopic labeling strategy using tandem mass tags (TMT) to identify and quantify tissue-derived proteins. In this approach, a tissue homogenate sample is used as a reference sample and labeled with one TMT, and then combined with plasma samples labeled with complementary TMTs. We used the Thermo Scientific TMT10plex Isobaric Mass Labeling Reagent Set for the experiments described here. To optimize our methodology, we focused on the putative use of kidney homogenate samples (cortex and medulla) to identify additional proteins in plasma samples. Proteins from tissue homogenate and plasma proteins were mixed in different ratios to assess the optimal protocol to maximize protein identification in plasma samples, and maximize reproducibility between replicate experiments. For example, a ratio of 5:1 (kidney tissue to plasma) contains 50 μg peptides from the kidney tissue homogenate for medulla and cortex (labeled with 126 and 127N reporter ions), and is combined with 10 μg peptides from each plasma sample (Table 1). Since it is anticipated that a significant fraction of peptides identified in this analysis will be present in much higher concentration in kidney homogenate compared to plasma, we always left one of the TMT reporter ion channels as a blank between the two kidney samples and the plasma samples to prevent interference of the reporter ions.

**Figure 1:**
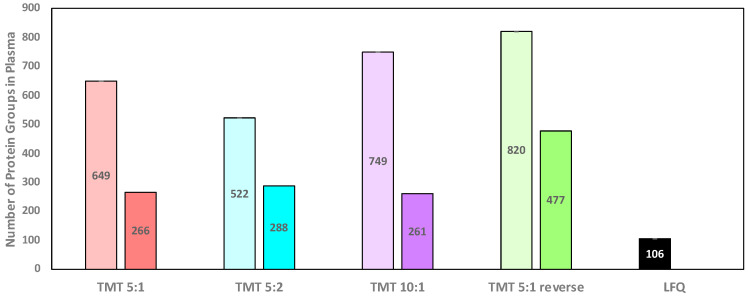
Number of proteins identified across all seven plasma samples for each methodology. The light-colored left bar depicts the total number of unique proteins identified across the two replicate experiments, and the darker right bar for each method depicts the number of proteins identified in both replicate experiments. Data can be found in Supplementary Tables.

As shown in Table 2, the use of a medulla and cortex reference sample significantly increases the number of proteins identified in the analysis for all kidney to plasma protein ratios tested (*p* = 7 × 10^−11^–2 × 10^−23^). The method TMT 5:1 identified on average 457 ± 19 proteins, the 10:1 ratio 499 ± 3 proteins, and TMT 5:2 401 ± 4 proteins. We also reversed the sample order in additional experiments where the cortex and medulla peptides were labeled with the highest molecular weight reporter ions (130C and 131). In this reversed experiment (method TMT 5:1 reverse), we identified on average 643 ± 16 proteins per plasma sample. Figure 1 summarizes the total number of proteins identified across all plasma samples and replicate experiments. Method TMT 5:1 reverse identifies the largest number of total proteins across all plasma samples (820), all other methods identify fewer proteins (649 for TMT 5:1, 522 for TMT 5:2, and 749 for TMT 10:1). 

When only proteins are considered that are identified in both replicate experiments, method TMT 5:1 reverse identified 477 proteins compared to 266, 288, and 261 for TMT 5:1. TMT 5:2, and TMT 10:1 experiments, respectively (Figure 1). Clearly, method TMT 5:1 reverse identified the most proteins in the plasma samples, the most proteins for each individual plasma sample, and the highest number of proteins identified in replicate experiments. It is important to note that the data highlight the depletion of abundant plasma proteins such as albumin. As shown in Supplementary Tables S2 and S3, the method TMT 5:1 reverse identified only 48 peptide spectral matches (1.17% of the total PSMs in the experiment) that match to albumin, a significant reduction for a protein that constitutes about 55% of the total plasma protein mass.

**Figure 2:**
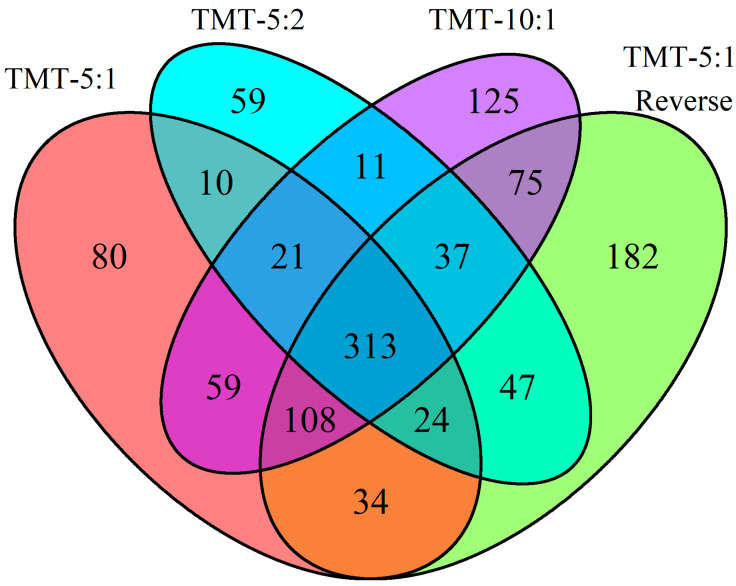
Venn diagram of proteins identified by each method from plasma samples. Protein IDs (see Supplementary Tables) were aligned and compared between methods.

As shown in Figure 2, 313 proteins are identified in all four different methods and experimental setups. TMT 5:1 reverse identified the highest number of unique proteins (182) that are not identified by any of the other methods. Of these 182 unique proteins, 117 are detected in the kidney samples with at least ten-fold higher intensity compared to the plasma samples, highlighting that the approach can detect low abundance plasma proteins that are more abundant in the tissue reference sample. There are 19 proteins found in the kidney samples that are only identified by the TMT 5:1 reverse. By comparison, all other methods find at most a single unique protein identified in kidney samples, highlighting that the TMT 5:1 reverse method identifies the largest number of proteins present in kidney and plasma. As expected, the overall reporter ion intensity for proteins detected only in the TMT 5:1 reverse method is also significantly lower than the intensity of the 313 proteins identified by all methods (*p* = 0.00165), suggesting that this method enhances the ability to detect low abundance proteins in plasma that may be derived from the kidney. 

The Human Protein Atlas (http://www.proteinatlas.org, accessed on 4 January 2023) identifies 452 genes that have at least four-fold higher mRNA expression levels in the kidney compared to any other tissues. We used this gene set to annotate the proteins identified by the different methods described above. About 50 of the proteins identified by the method TMT 5:1 reverse are annotated as kidney-enriched. These five proteins (ACAA1, LGALS2, SLC4A4, SLC2A6, and TPMT) are only detected by the method TMT 5:1 reverse. All of these proteins are expressed in the proximal tubules of the kidney. In contrast, only one protein identified by the LFQ analysis of the plasma samples (GPX3) is included in the list of kidney-enriched proteins. This annotation demonstrates that a high proportion of the additional proteins identified by the TMT labeling approach are kidney-enriched, and may serve as potential biomarkers for kidney function or dysfunction. For example, the gene solute carrier family 4 member 4 (SLC4A4) encodes a sodium bicarbonate cotransporter, and the gene has been associated with renal tubular acidosis [23].

**Figure 3:**
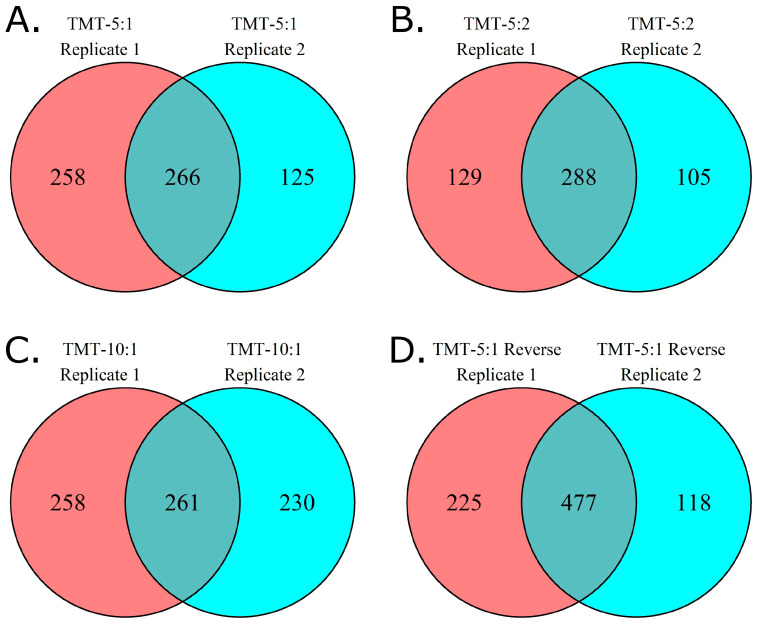
Protein IDs shared between replicates of each method. Each experiment was run twice, using seven plasma samples. Unique protein IDs were compared for all seven samples for each method.

The reproducibility of protein identification between replicate experiments is variable across methods. When we compare the proteins identified across all seven plasma samples for each of the two replicate experiments (Figure 3), 58% of the proteins identified in method TMT 5:1 reverse were shared between replicates, similar to the 55% shared between replicates for TMT 5:2. However, the other methods share fewer proteins between replicates (41% for TMT 5:1, 35% for TMT 10:1).

Taken together, our data demonstrate that the use of a kidney reference sample in a TMT quantitative proteomics analysis of plasma samples significantly increases the number of proteins identified as well as the number of kidney-enriched proteins. A ratio of 5:1 with the kidney reference sample labeled with the heaviest reporter ion (TMT 5:1 reverse) results in the largest number of unique proteins identified, the highest number of proteins per plasma sample, and the results are most reproducible between replicate experiments, suggesting that this is the best experimental design to enhance the discovery of putative tissue leakage proteins in plasma samples using TMT-labeled proteomics mass spectrometry.

## 4. Discussion

Analysis of tissue leakage proteins in plasma samples using untargeted mass spectrometry approaches remains a challenge. Only a small fraction of proteins account for over 95% of the total protein content in plasma, and these highly abundant plasma proteins impede effective detection and quantification of the remaining, less abundant proteins (REF). However, low abundance proteins may oftentimes be the very proteins that are indicative of disease processes in particular tissues and organs. As tissue deteriorates, tissue-derived proteins are released into the blood stream in small quantities, particularly during early stages of disease development. Such proteins are of particular interest as potential disease biomarkers, especially if they are likely released as part of the disease process. However, they remain challenging to identify and quantify. While some recent and more sensitive (e.g., targeted) mass spectrometry approaches improve the detection and quantification of low abundance proteins, linking them to particular pathophysiological processes in tissues or organs remains difficult. 

Recent adaptations of tandem mass tag (TMT) labeling in proteomics have shown that a TMT labeling approach can be used to effectively identify proteins at very low abundance if the analysis also includes a reference sample. In single cell proteomics, the SCoPE-MS approach uses a reference sample of proteins extracted from several hundred cells for protein identification in MS1 and MS2, and then uses proteins labeled with other TMT reporters to quantify those proteins in individual cells [21]. In our study, we adapted this approach to the analysis of plasma samples, using protein extracted from a tissue homogenate as a reference sample. In this study, we used kidney homogenates as a reference to analyze plasma samples from a recently published study on sodium exposure and hypertension in baboons. Both sodium exposure and hypertension have been reported to affect kidney function, and gene expression analysis of kidney biopsy samples has revealed substantial changes in the kidney in response to even a relatively short sodium exposure [22].

Our mass spectrometry data clearly demonstrate that the use of a kidney protein extract as a reference sample in a TMT experiment dramatically increases the number of proteins identified in plasma samples. The optimal methodology uses the heaviest TMT reporter ion for the reference sample. This likely minimizes reporter ion interference with other reporter ion channels, and leads to the identification and quantification of 820 proteins in these plasma samples. A substantial fraction of the proteins only identified by the optimal methodology (that we named TMT 5:1 reverse) is also identified in the kidney homogenate, but is much more abundant in the kidney, supporting our hypothesis that this approach will identify kidney proteins (leakage proteins) that are much less abundant in plasma. 

Obviously, not all proteins identified in our analysis are exclusively expressed in the kidney. In fact, analysis of GTEx data suggests that some proteins are significantly enriched in particular tissues, but very few are exclusively expressed in one or only few tissues [24]. As we discuss above, our optimized methodology identified 50 proteins that are defined as kidney-enriched in the Human Protein Atlas database. Therefore, the methodology we present here clearly not only identifies more proteins in plasma samples compared to standard label-free quantification approaches, but also a substantial fraction of the proteins identified in plasma using this novel approach are likely derived from kidney tissue, and therefore may be useful as markers for kidney function or dysfunction. 

Our analysis only included a small set of four animals from the initial study. Given our sample size and the short duration of the experiment, we would not expect to detect any proteins that are associated with sodium exposure or blood pressure variation at this point. Clearly, future studies will explore the usefulness of the approach described here to identify sets of protein biomarkers that are associated with particular kidney diseases. Given a set of over 800 proteins that can be monitored and quantified using our TMT labeling approach, this significantly increases the probability of finding kidney-derived biomarkers using this untargeted mass spectrometry approach.

There are other technologies being promoted for plasma proteome analysis such as Somascan, an aptamer-based affinity analysis [25,26], and Proteograph, a nanoparticle-based enrichment strategy [27,28] that promise to increase the ability to identify and quantify low abundance proteins in plasma. Both methods report a much larger number of quantified proteins compared to our methodology. However, these approaches do not “preselect” for proteins that are also present in the kidney, so it remains to be seen whether the sheer number of quantifiable proteins will yield better biomarker signatures using these approaches. Any protein discovered using our approach is expressed in the kidney (since it is identified using kidney homogenate), and therefore may be related to organ-specific pathologies when found at elevated levels in plasma.

Overall, our pilot work has allowed us to quantify proteins that are in low abundance and difficult to detect using standard label-free proteomics strategies and even more difficult to link back to an organ or tissue. The innovation of introducing TMT-labels with tissue reference samples in multiplexing enables us to explore the biological significance of specific tissue proteins in plasma, and extend the number of putative biomarkers for diseases. Clearly, this work can be performed with reference samples from other disease tissues as well (liver, heart, lung, etc.,), opening the door to future discoveries of early disease biomarker signatures that are tissue- or organ-specific and link to the underlying pathophysiology of a disease.

## Figures and Tables

**Table 1:** Description of the TMT mixtures of kidney and plasma proteins for the various methods tested in this study.

Method Name	Protein Amount Kidney	Protein Amount Plasma	TMT Reporter for Kidney Samples
TMT 5:1	50 μg	10 μg	126 + 127N
TMT 5:2	50 μg	20 μg	126 + 127N
TMT 10:1	100 μg	10 μg	126 + 127N
TMT 5:1 reverse	50 μg	10 μg	130C + 131

**Table 2:** Number of protein groups identified in the analysis of each sample. Detailed results for each experiment can be found in the Supplementary Tables S2–S11.

Sample	TMT 5:1 R1	TMT 5:1 R2	TMT 10:1 R1	TMT 10:1 R2	TMT 5:2 R1	TMT 5:2 R2	TMT 5:1rev R1	TMT 5:1rev R2	LFQ
15286 cortex	392	524	491	522	417	389	702	589	489
15286 medulla	392	524	491	515	411	379	704	597	1780
15562 6wk plasma	389	524	491	514	416	390	702	583	135
15562 12wk plasma	386	524	487	498	415	383	697	582	108
15286 6wk plasma	390	523	489	509	416	389	698	589	79
15286 12wk plasma	391	524	491	518	416	391	700	593	85
15122 6wk plasma	391	523	491	498	411	384	700	586	120
15122 12wk plasma	391	523	491	507	415	387	697	586	103
17174 6wk plasma	390	524	491	508	416	388	701	593	109

* R1, R2: Replicate experiments; LFQ: Label-free quantification.

## Data Availability

All mass spectrometry proteomics data are available at PRIDE (Accession number PXD039430). All analyzed data are available in the Supplementary Tables.

## Supplementary Materials

The following supporting information can be downloaded at: https://www.mdpi.com/article/10.3390/biom13020215/s1. Supplementary Tables S1–S11. Table S1: TMT channels; Table S2: 5 × 1rev Replicate1 MQ; Table S3: 5 × 1rev Replicate2 MQ; Table S4: 5 × 1 Replicate1 MQ; Table S5: 5 × 1 Replicate2 MQ; Table S6: 5 × 2 Replicate1 MQ; Table S7: 5 × 2 Replicate2 MQ; Table S8: 10 × 1 Replicate1 MQ; Table S9: 10 × 1 Replicate2 MQ; Table S10: Plasma LFQ MQ; Table S11: Kidney LFQ MQ.

## References

[1] Olivier M., Asmis R., Hawkins G.A., Howard T.D., Cox L.A. (2019). The Need for Multi-Omics Biomarker Signatures in Precision Medicine. Int. J. Mol. Sci..

[2] Arora A., Sharma P. (2012). Non-invasive Diagnosis of Fibrosis in Non-alcoholic Fatty Liver Disease. J. Clin. Exp. Hepatol..

[3] Chiu K.-W., Chen Y.-S., De Villa V.H., Wang C.-C., Eng H.-L., Wang S.-H., Liu P.-P., Jawan B., Huang T.-L., Cheng Y.-F. (2005). Characterization of liver enzymes on living related liver transplantation patients with acute rejection. Hepato Gastroenterol..

[4] Katzke V., Johnson T., Sookthai D., Hüsing A., Kühn T., Kaaks R. (2020). Circulating liver enzymes and risks of chronic diseases and mortality in the prospective EPIC-Heidelberg case-cohort study. BMJ Open.

[5] Scutt P., Ban L., Card T., Crooks C.J., Guha N., West J., Morling J.R. (2022). Liver blood marker testing in UK primary care: A UK wide cohort study, 2004–2016. BMJ Open.

[6] Smid V. (2022). Liver tests. Cas. Lek. Cesk..

[7] Zhang L., Ma X., Jiang Z., Zhang K., Zhang M., Li Y., Zhao X., Xiong H. (2015). Liver enzymes and metabolic syndrome: A large-scale case-control study. Oncotarget.

[8] Chan D., Ng L.L. (2010). Biomarkers in acute myocardial infarction. BMC Med..

[9] Duque-Ossa L., García-Ferrera B., Reyes-Retana J. (2021). Troponin I as a Biomarker for Early Detection of Acute Myocardial Infarction. Curr. Probl. Cardiol..

[10] Perna E., Canella J.P.C., Coronel M.L., Macin S.M. (2016). The predictive value of plasma biomarkers in discharged heart failure patients: Role of troponin I/T. Minerva Cardioangiol..

[11] Baker E.S., Liu T., Petyuk V.A., Burnum-Johnson K.E., Ibrahim Y.M., Anderson G.A., Smith R.D. (2012). Mass spectrometry for translational proteomics: Progress and clinical implications. Genome Med..

[12] Geyer P.E., Holdt L.M., Teupser D., Mann M. (2017). Revisiting biomarker discovery by plasma proteomics. Mol. Syst. Biol..

[13] Cox J., Mann M. (2008). MaxQuant enables high peptide identification rates, individualized p.p.b.-range mass accuracies and proteome-wide protein quantification. Nat. Biotechnol..

[14] Eng J.K., McCormack A.L., Yates J.R. (1994). An approach to correlate tandem mass spectral data of peptides with amino acid sequences in a protein database. J. Am. Soc. Mass Spectrom..

[15] Jemal M., Ouyang Z., Powell M.L. (2000). Direct-injection LC–MS–MS method for high-throughput simultaneous quantitation of simvastatin and simvastatin acid in human plasma. J. Pharm. Biomed. Anal..

[16] Aebersold R., Mann M. (2003). Mass spectrometry-based proteomics. Nature.

[17] Cao X., Sandberg A., Araújo J.E., Cvetkovski F., Berglund E., Eriksson L.E., Pernemalm M. (2021). Evaluation of Spin Columns for Human Plasma Depletion to Facilitate MS-Based Proteomics Analysis of Plasma. J. Proteome Res..

[18] Chahrour O., Cobice D., Malone J. (2015). Stable isotope labelling methods in mass spectrometry-based quantitative proteomics. J. Pharm. Biomed. Anal..

[19] Moulder R., Bhosale S.D., Goodlett D.R., Lahesmaa R. (2018). Analysis of the plasma proteome using iTRAQ and TMT-based Isobaric labeling. Mass Spectrom. Rev..

[20] Rauniyar N., Yates J.R. (2014). Isobaric Labeling-Based Relative Quantification in Shotgun Proteomics. J. Proteome Res..

[21] Budnik B., Levy E., Harmange G., Slavov N. (2018). SCoPE-MS: Mass spectrometry of single mammalian cells quantifies proteome heterogeneity during cell differentiation. Genome. Biol..

[22] Riojas A.M., Reeves K.D., Shade R.E., Puppala S.R., Christensen C.L., Birnbaum S., Glenn J.P., Li C., Shaltout H., Hall-Ursone S. (2022). Blood pressure and the kidney cortex transcriptome response to high-sodium diet challenge in female nonhuman primates. Physiol. Genom..

[23] Parker M.D., Qin X., Williamson R.C., Toye A.M., Boron W.F. (2012). HCO_3_^−^-independent conductance with a mutant Na^+^/HCO_3_^−^ cotransporter (SLC4A4) in a case of proximal renal tubular acidosis with hypokalaemic paralysis. J Physiol..

[24] Jiang L., Wang M., Lin S., Jian R., Li X., Chan J., Dong G., Fang H., Robinson A.E., Snyder M.P. (2020). A Quantitative Proteome Map of the Human Body. Cell.

[25] Candia J., Cheung F., Kotliarov Y., Fantoni G., Sellers B., Griesman T., Huang J., Stuccio S., Zingone A., Ryan B.M. (2017). Assessment of Variability in the SOMAscan Assay. Sci. Rep..

[26] Candia J., Daya G.N., Tanaka T., Ferrucci L., Walker K.A. (2022). Assessment of variability in the plasma 7k SomaScan proteomics assay. Sci. Rep..

[27] Ferdosi S., Stukalov A., Hasan M., Tangeysh B., Brown T., Wang T., Elgierari E., Zhao X., Huang Y., Brittany A. (2022). Enhanced Competition at the Nano-Bio Interface Enables Comprehensive Characterization of Protein Corona Dynamics and Deep Coverage of Proteomes. Adv. Mater..

[28] Liu Y., Wang J., Xiong Q., Hornburg D., Tao W., Farokhzad O.C. (2020). Nano–Bio Interactions in Cancer: From Therapeutics Delivery to Early Detection. Accounts Chem. Res..

